# Supplementary material for: ProRefiner: an entropy-based refining strategy for inverse protein folding with global graph attention
Source: Nat Commun. 2023 Nov 16;14:7434. doi: 10.1038/s41467-023-43166-6 (PMC10654420; doi:10.1038/s41467-023-43166-6)
Supplement: Supplementary file 1 — Supplementary Information [file 41467_2023_43166_MOESM1_ESM.pdf]

Supplementary Information for ProRefiner: An Entropy-based Refining Strategy for Inverse Protein Folding with Global Graph Attention

Xinyi Zhou[1], Guangyong Chen[2*], Junjie Ye[3], Ercheng Wang[2,4], Jun Zhang[5], Cong Mao[5], Zhanwei Li[2], Jianye Hao[3], Xingxu Huang[2], Jin Tang[2] and Pheng Ann Heng[1,2]

[1]Department of Computer Science and Engineering, The Chinese University of Hong Kong, Central Ave, Hong Kong, China.
[2]Zhejiang Lab, Kechuang Avenue, Hangzhou, China.
[3]Noah's Ark Lab, Huawei, Shenzhen, China.
[4]College of Pharmaceutical Sciences, Zhejiang University, Hangzhou, China.
[5]State Key Laboratory of Reproductive Medicine, Nanjing Medical University, Nanjing, China.

*Corresponding author(s). E-mail(s): gychen@zhejianglab.com;

The PDF file includes:

Supplementary Table 1 to Supplementary Table 6

Supplementary Results

Model Perplexity

Perplexity is a useful and commonly reported metric for evaluating autoregressive language models. Since autoregressive models generate a token based on all previous tokens, the probability of a sequence could be computed by the chain rule, and the model perplexity could be calculated from the probability of the dataset. Previous inverse folding models generally fall into this category. However, our ProRefiner belongs to the class of masked language models, such as BERT, which are discriminative models trained to predict missing tokens in a given input sequence, and as such, the chain rule does not apply [1, 2], making perplexity ill-defined for these models [3, 4].

Therefore, we compute the pseudo-perplexity developed for masked language models following [4], which is not theoretically well justified but can still approximate sequence probabilities. In Supplementary Table 1, ProteinMPNN and ESM-IF1 in the first group are trained on their customized dataset and other three models are trained on CATH training set.

Supplementary Table 1 Perplexity of models. Perplexity of ProRefiner is pseudo-perplexity computed following [4].

Model	CATH	TS50	Latest PDB	EnzBench	BR_EnzBench
ProteinMPNN	5.41	5.12	4.39	4.50	4.92
ESM-IF1	3.99	3.43	2.82	2.95	3.39
GVP-GNN	5.44	4.94	4.43	4.43	5.13
ProteinMPNN-C	5.21	4.52	3.83	3.89	4.47
ProRefiner	3.90	3.62	3.10	3.15	3.67

Sequence Refining on Partial Sequence Design

We have showcased the application of ProRefiner as an add-on module for refining base models' sequences within the context of entire sequence design. However, it is important to note that this application is not limited to entire sequence design. In this section, we apply the proposed entropy-based mask and subsequently refinement on partial sequence design. To demonstrate this, we conduct experiments with base model ESM-IF1. Supplementary Table 2 presents the performance of ESM-IF1 and ProRefiner + ESM-IF1 on partial sequence design. Note that the first group of models in the table is trained on CATH training split while ESM-IF1 is trained on a significantly larger dataset. The results demonstrate that while ESM-IF1 achieves high recovery and nssr on its own, applying the proposed refinement by ProRefiner can further enhance performance.

Supplementary Table 2 Median sequence recovery rates and nssr scores on EnzBench and BR_EnzBench. Data in brackets reports the 95% confidence interval of the median, estimated from 10,000 bootstrap samples. The three models in the first group are trained on CATH training split, while ESM-IF1 is trained on a larger customized dataset.

	EnzBench n = 51		BR_EnzBench n = 320	
	Recovery %	nssr %	Recovery %	nssr %
GVP-GNN	$41.38_{[36.36,42.86]}$	$57.89_{[55.00,63.16]}$	$29.41_{[27.27,31.58]}$	$47.83_{[47.37,52.17]}$
ProteinMPNN-C	$52.00_{[50.00,59.09]}$	$70.00_{[65.00,77.78]}$	$40.91_{[40.00,42.48]}$	$60.00_{[59.09,60.87]}$
ProRefiner	$57.89_{[55.00,63.64]}$	$73.68_{[70.59,78.26]}$	$43.48_{[41.64,44.44]}$	$60.87_{[59.09,63.64]}$
ESM-IF1	$60.71_{[57.89,66.67]}$	$77.27_{[68.42,81.25]}$	$52.63_{[50.00,56.52]}$	$72.00_{[69.57,73.91]}$
ProRefiner+ESM-IF1	$64.29_{[61.11,72.73]}$	$81.82_{[75.00,85.71]}$	$54.55_{[50.00,59.09]}$	$72.00_{[69.57,72.73]}$

Target Structure Recovery

We investigate how well the designed sequences can fold into target structures. We conduct structural evaluation on TS50 and CATH datasets for entire sequence design and EnzBench dataset for partial sequence design. For partial sequence design, we additionally report the performance of ESM-IF1 and ProRefiner + ESM-IF1 as in previous section. We predict the structures of designed sequences and report their TM-score and RMSD compared to target structures, and the pLDDT computed by the folding algorithm. Due to limited resources, we utilized Alphafold2, a computationally intensive and resource-demanding method, to fold the two relatively small datasets, TS50 and EnzBench. We run Alphafold2 with both MSA and searched templates and select the top-ranked structures as our predicted structures. Meanwhile, we employ ESMFold [5] for folding the largest dataset, CATH. We run 3 recycles for each sequence. Results are reported in Supplementary Table 3 ∼ Supplementary Table 5.

We observe that the sequence recovery and structural recovery of the model are not positively correlated. While some methods, such as ESM-IF1 and ProRefiner, achieves significantly higher sequence recovery than other methods, their improvement in structure recovery is limited. Meanwhile, the discrepancies in structure recovery among the models are significantly smaller than those in sequence recovery. Models exhibit similar levels of structure recovery, despite the variations in their performance of sequence prediction accuracy. We believe that this may be due to the fact that the recovery of target structures is a more complex metric than sequence recovery. It is influenced by a variety of factors beyond the accuracy of the predicted sequence, including the accuracy of the folding algorithm itself. We also speculate that prior models may have hit an accuracy ceiling for structure recovery when relying solely on optimizing sequence recovery as training objective, as evidenced by their similar structure recovery performance. Overcoming this may require directly optimizing structure recovery in an end-to-end framework. We plan to investigate the problem and explore this direction in future research.

Supplementary Table 3 The median TM-score, RMSD and pLDDT for predicted structures on dataset TS50 (n = 50). Data in brackets reports the 95% confidence interval of the median, estimated from 10,000 bootstrap samples. Alphafold2 is used to predict the structures of sequences designed by models.

Model	TM-score ↑	RMSD ↓	pLDDT ↑
ProteinMPNN	$0.971_{[0.962,0.977]}$	$0.948_{[0.693,1.136]}$	$95.163_{[94.222,95.924]}$
ProRefiner + ProteinMPNN	$0.967_{[0.960,0.973]}$	$0.940_{[0.809,1.091]}$	$94.679_{[94.184,95.584]}$
ProteinMPNN-C	$0.969_{[0.961,0.973]}$	$0.997_{[0.746,1.223]}$	$94.716_{[94.291,95.295]}$
ProRefiner + ProteinMPNN-C	$0.971_{[0.960,0.976]}$	$0.886_{[0.777,1.156]}$	$95.362_{[94.240,95.553]}$
ESM-IF1	$0.974_{[0.966,0.981]}$	$0.838_{[0.722,1.116]}$	$95.186_{[94.253,96.045]}$
ProRefiner + ESM-IF1	$0.972_{[0.963,0.978]}$	$0.873_{[0.754,1.152]}$	$94.853_{[94.147,95.688]}$

Supplementary Table 4 The median TM-score, RMSD and pLDDT for predicted structures on dataset CATH (n = 1,120). Data in brackets reports the 95% confidence interval of the median, estimated from 10,000 bootstrap samples. ESMFold is used to predict the structures of sequences designed by models.

Model	TM-score ↑	RMSD ↓	pLDDT ↑
ProteinMPNN	$0.861_{[0.852,0.867]}$	$10.851_{[10.099,11.500]}$	$82.315_{[81.909,82.698]}$
ProRefiner + ProteinMPNN	$0.848_{[0.840,0.858]}$	$11.151_{[10.286,11.686]}$	$82.069_{[81.527,82.443]}$
ProteinMPNN-C	$0.845_{[0.836,0.852]}$	$11.142_{[10.462,11.836]}$	$80.574_{[80.141,81.031]}$
ProRefiner + ProteinMPNN-C	$0.848_{[0.835,0.855]}$	$11.026_{[10.283,11.762]}$	$80.996_{[80.469,81.585]}$
ESM-IF1	$0.854_{[0.846,0.862]}$	$10.827_{[10.242,11.629]}$	$81.693_{[81.224,82.230]}$
ProRefiner + ESM-IF1	$0.852_{[0.847,0.862]}$	$10.897_{[10.324,11.664]}$	$81.976_{[81.286,82.476]}$

Supplementary Table 5 The median TM-score, RMSD and pLDDT for predicted structures on dataset EnzBench (n = 51). Data in brackets reports the 95% confidence interval of the median, estimated from 10,000 bootstrap samples. Alphafold2 is used to predict the structures of sequences designed by models.

Model	TM-score ↑	RMSD ↓	pLDDT ↑
GVP-GNN	$0.977_{[0.964,0.983]}$	$0.906_{[0.622,1.570]}$	$95.365_{[93.981,96.326]}$
ProteinMPNN-C	$0.983_{[0.971,0.990]}$	$0.812_{[0.528,1.171]}$	$95.598_{[94.775,96.890]}$
ProRefiner	$0.982_{[0.972,0.988]}$	$0.848_{[0.608,1.143]}$	$95.974_{[94.742,96.466]}$
ESM-IF1	$0.981_{[0.971,0.989]}$	$0.833_{[0.523,1.260]}$	$96.118_{[94.871,96.695]}$
ProRefiner + ESM-IF1	$0.983_{[0.972,0.990]}$	$0.804_{[0.517,1.015]}$	$96.112_{[94.869,96.801]}$

Comparison with Vanilla Global Attention

In addition to the Inverse Protein Folding task, we conduct experiments on the following evaluation tasks to compare the predictive performance of the proposed memory-efficient global attention and the vanilla self-attention. We still use ProRefiner to denote the models with the memory-efficient global attention layers, and ProRefiner - PsFeat to denote the models using the original global attention layers.

- Relative Solvent Accessibility (RSA). We train models to predict the relative solvent accessibility of residues. Both models have 4 attention layers and model inputs are protein sequences and backbone structures. Input residue

graphs are constructed as in Inverse Protein Folding. A scalar between 0 and 1 is output for each residue through a Sigmoid layer. We employ DSSP program [6] to calculate the RSA of each residue in CATH dataset, and evaluate on its testing split. Evaluation metric is the Pearson correlation coefficient between predicted and actual RSA.

- Ligand Binding Affinity (LBA) We predict the binding affinity of ligands to their corresponding proteins based on the co-crystallized structure of the protein-ligand complex. Both models have 4 attention layers. Model inputs are the atoms of the pocket and ligand. An atom graph is constructed for each protein-ligand pair by connecting each atom with its 64 nearest neighbors. The models are trained to predict $-\log(K)$, where K is the binding affinity in Molar units. We employ the LBA dataset from [7] split by 30% sequence identity. Evaluation metric is the Pearson correlation coefficient between predicted and actual affinity.
- Small Molecule Properties (SMP) We predict two properties of small molecules: dipole moment (μ) and zero point vibrational energy (ZPVE). Both models have 4 attention layers. Model inputs are the atoms of molecules. Two atoms are connected in a graph if their distance is less than 4.5 Å. The models are trained to predict the corresponding property of the molecule. We employ the SMP dataset from [7] and use mean absolute error as metric.

Supplementary Table 6 Performance of ProRefiner and ProRefiner - PsFeat on 4 tasks. The metric for RSA and LBA is Pearson correlation between the predicted values and true values of all samples in the datasets (higher the better). The metric for SMP tasks is mean absolute error (lower the better). Data in brackets reports the 95% confidence interval of the mean, estimated from 10,000 bootstrap samples.

	RSA	LBA	SMP - μ	SMP - ZPVE
	n = 172,106	n = 490	n = 12,943	n = 12,943
ProRefiner - Psfeat	0.897	0.542	$0.153_{[0.150,0.156]}$	$4.626e-4_{[4.550e-4,4.705e-4]}$
ProRefiner	0.888	0.539	$0.166_{[0.162,0.169]}$	$6.112e-4_{[5.989e-4,6.238e-4]}$

According to results in Supplementary Table 6, the performance of ProRefiner is slightly worse but remains generally comparable to that of ProRefiner - PsFeat on all tasks.

Supplementary References

[1] Devlin J, Chang MW, Lee K, Toutanova K. Bert: Pre-training of deep bidirectional transformers for language understanding. arXiv preprint arXiv:181004805. 2018;.

[2] Rezaee M, Darvish K, Kebe GY, Ferraro F. Discriminative and generative transformer-based models for situation entity classification. arXiv preprint arXiv:210907434. 2021;.

[3] Wang A, Cho K. BERT has a mouth, and it must speak: BERT as a Markov random field language model. arXiv preprint arXiv:190204094. 2019;.

[4] Salazar J, Liang D, Nguyen TQ, Kirchhoff K. Masked language model scoring. arXiv preprint arXiv:191014659. 2019;.

[5] Lin Z, Akin H, Rao R, Hie B, Zhu Z, Lu W, et al. Language models of protein sequences at the scale of evolution enable accurate structure prediction. bioRxiv. 2022;.

[6] Kabsch W, Sander C. Dictionary of protein secondary structure: pattern recognition of hydrogen-bonded and geometrical features. Biopolymers: Original Research on Biomolecules. 1983;22(12):2577–2637.

[7] Townshend RJ, Vögele M, Suriana P, Derry A, Powers A, Laloudakis Y, et al. Atom3d: Tasks on molecules in three dimensions. arXiv preprint arXiv:201204035. 2020;.
