# Supplementary material for: ProRefiner: an entropy-based refining strategy for inverse protein folding with global graph attention
Source: Nat Commun. 2023 Nov 16;14:7434. doi: 10.1038/s41467-023-43166-6 (PMC10654420; doi:10.1038/s41467-023-43166-6)
Supplement: Supplementary file 3 — Reporting Summary [file 41467_2023_43166_MOESM3_ESM.pdf]

nature portfolio

Corresponding author(s): Guangyong Chen

Last updated by author(s): Oct 5, 2023

Reporting Summary

Nature Portfolio wishes to improve the reproducibility of the work that we publish. This form provides structure for consistency and transparency in reporting. For further information on Nature Portfolio policies, see our Editorial Policies and the Editorial Policy Checklist.

Statistics

For all statistical analyses, confirm that the following items are present in the figure legend, table legend, main text, or Methods section.

n/a	Confirmed	
☐	☒	The exact sample size (*n*) for each experimental group/condition, given as a discrete number and unit of measurement
☐	☒	A statement on whether measurements were taken from distinct samples or whether the same sample was measured repeatedly
☐	☒	The statistical test(s) used AND whether they are one- or two-sided<br>*Only common tests should be described solely by name; describe more complex techniques in the Methods section.*
☒	☐	A description of all covariates tested
☒	☐	A description of any assumptions or corrections, such as tests of normality and adjustment for multiple comparisons
☐	☒	A full description of the statistical parameters including central tendency (e.g. means) or other basic estimates (e.g. regression coefficient) AND variation (e.g. standard deviation) or associated estimates of uncertainty (e.g. confidence intervals)
☐	☒	For null hypothesis testing, the test statistic (e.g. *F*, *t*, *r*) with confidence intervals, effect sizes, degrees of freedom and *P* value noted<br>*Give P values as exact values whenever suitable.*
☒	☐	For Bayesian analysis, information on the choice of priors and Markov chain Monte Carlo settings
☒	☐	For hierarchical and complex designs, identification of the appropriate level for tests and full reporting of outcomes
☐	☒	Estimates of effect sizes (e.g. Cohen's *d*, Pearson's *r*), indicating how they were calculated

Our web collection on statistics for biologists contains articles on many of the points above.

Software and code

Policy information about availability of computer code

Data collection	For public datasets CATH (v4.2), TS50 (v2.0), EnzBench (from Rosetta v3.13), BR_EnzBench, Ligand Binding Affinity dataset (v0.1) and Small Molecule Properties (v0.1) dataset we directly use the published data.<br>For Latest PDB dataset, we use custom code to collect the PDB files from https://www.rcsb.org/ and data processing used Biopython v1.80 (https://biopython.org/).
Data analysis	TM-score and RMSD calculation used TM-score v20220415 from https://zhanggroup.org/TM-score/.<br>Mantel test used mantel v2.2.0 from https://github.com/jwcarr/mantel.<br>Other data analysis used Python v3.9.16 (https://www.python.org/), NumPy v1.24.3 (https://numpy.org/), SciPy v1.10.1 (https://scipy.org/), PyTorch v1.13.0 (https://pytorch.org/), pandas v2.0.2 (https://pandas.pydata.org/), Matplotlib v3.7.1 (https://matplotlib.org/), seaborn v0.12.2 (https://seaborn.pydata.org/) and Biopython v1.80 (https://biopython.org/).<br>Structure visualizations were created in ChimeraX v1.6.1 (https://www.cgl.ucsf.edu/chimerax/).<br>Indel analysis used CRISPResso2 (https://github.com/pinellolab/CRISPResso2).<br>The code developed in this manuscript is deposited in Colab (https://colab.research.google.com/drive/1a6VW-BB0twEwL65sE_dUAM42wdSm6RZp?usp=sharing), Code Ocean (https://codeocean.com/capsule/9492154/tree) and Github (https://zenodo.org/records/10030882).

For manuscripts utilizing custom algorithms or software that are central to the research but not yet described in published literature, software must be made available to editors and reviewers. We strongly encourage code deposition in a community repository (e.g. GitHub). See the Nature Portfolio guidelines for submitting code & software for further information.

Data

Policy information about availability of data

All manuscripts must include a data availability statement. This statement should provide the following information, where applicable:

- Accession codes, unique identifiers, or web links for publicly available datasets
- A description of any restrictions on data availability
- For clinical datasets or third party data, please ensure that the statement adheres to our policy

CATH dataset (v4.2) is available at: http://people.csail.mit.edu/ingraham/graph-protein-design/data/.
TS50 dataset (v2.0) is available at: https://zenodo.org/record/6650679#.ZDJJNhVByhY.
EnzBench is available as part of the standard Rosetta package (v3.13) which could be downloaded from https://www.rosettacommons.org/software/license-and-download with a license.
BR_EnzBench is provided by and available from the paper https://doi.org/10.1371/journal.pcbi.1005600.
Latest PDB dataset is available at https://drive.google.com/file/d/1Ate5I0Hz5GwzOJN4sQL_RrDUkjxMJZ0u/view?usp=sharing.
Ligand Binding Affinity dataset (v0.1) is available at https://zenodo.org/record/4914718.
Small Molecule Properties dataset (v0.1) is available at https://zenodo.org/record/4911142.
Source data are provided with this paper and through Figshare (https://doi.org/10.6084/m9.figshare.23913147).

Research involving human participants, their data, or biological material

Policy information about studies with human participants or human data. See also policy information about sex, gender (identity/presentation), and sexual orientation and race, ethnicity and racism.

Reporting on sex and gender	Not applicable; the research does not involve human participants.
Reporting on race, ethnicity, or other socially relevant groupings	Not applicable; the research does not involve human participants.
Population characteristics	Not applicable; the research does not involve human participants.
Recruitment	Not applicable; the research does not involve human participants.
Ethics oversight	Not applicable; the research does not involve human participants.

Note that full information on the approval of the study protocol must also be provided in the manuscript.

Field-specific reporting

Please select the one below that is the best fit for your research. If you are not sure, read the appropriate sections before making your selection.

☒ Life sciences ☐ Behavioural & social sciences ☐ Ecological, evolutionary & environmental sciences

For a reference copy of the document with all sections, see nature.com/documents/nr-reporting-summary-flat.pdf

Life sciences study design

All studies must disclose on these points even when the disclosure is negative.

Sample size	No statistical method was used to predetermine sample size; the methods are evaluated on the full CATH test set, TS50, Latest PDB, EnzBench and BR_EnzBench dataset.
Data exclusions	We excluded multichain structures and structures of a length more than 500 or resolution > 2.5 Å for Latest PDB dataset. No data were excluded from the analysis for other benchmarks.
Replication	All genome editing attempts were performed with at least three biological repeats. All attempts at reproducibility were successful and standard deviations were in the expected ranges.
Randomization	Not applicable; we are not making a comparison between different groups.
Blinding	Not applicable; we are not making a comparison between different groups.

Reporting for specific materials, systems and methods

We require information from authors about some types of materials, experimental systems and methods used in many studies. Here, indicate whether each material, system or method listed is relevant to your study. If you are not sure if a list item applies to your research, read the appropriate section before selecting a response.

Materials & experimental systems

n/a	Involved in the study
☒	☐ Antibodies
☐	☒ Eukaryotic cell lines
☒	☐ Palaeontology and archaeology
☒	☐ Animals and other organisms
☒	☐ Clinical data
☒	☐ Dual use research of concern
☒	☐ Plants

Methods

n/a	Involved in the study
☒	☐ ChIP-seq
☒	☐ Flow cytometry
☒	☐ MRI-based neuroimaging

Eukaryotic cell lines

Policy information about cell lines and Sex and Gender in Research

Cell line source(s)	293T cells have been purchased from ATCC.
Authentication	293T cells have been authenticated by ligth microscopy but not by additional methods.
Mycoplasma contamination	293T cells were tested monthly for mycoplasma contamination and all cell lines were tested negative for mycoplasma.
Commonly misidentified lines (See ICLAC register)	No cell lines used in this paper are listed in the database of commonly misidentified cell lines maintained by ICLAC.
